# Association of Traumatic Brain Injury With and Without Loss of Consciousness With Neuropathologic Outcomes in Community-Dwelling Older Persons

**DOI:** 10.1001/jamanetworkopen.2022.9311

**Published:** 2022-04-27

**Authors:** Sonal Agrawal, Sue E. Leurgans, Bryan D. James, Lisa L. Barnes, Rupal I. Mehta, Kristen Dams-O’Connor, Jesse Mez, David A. Bennett, Julie A. Schneider

**Affiliations:** 1 Rush Alzheimer’s Disease Center, Rush University Medical Center, Chicago, Illinois; 2 Department of Pathology, Rush University Medical Center, Chicago, Illinois; 3 Department of Neurological Sciences, Rush University Medical Center, Chicago, Illinois; 4 Department of Behavioral Sciences, Rush University Medical Center, Chicago, Illinois; 5 Department of Rehabilitation and Human Performance, Mt Sinai School of Medicine, New York, New York; 6 Department of Neurology, Mt Sinai School of Medicine, New York, New York; 7 Boston University Alzheimer’s Disease Center, Boston University School of Medicine, Boston, Massachusetts

## Abstract

**Importance:**

A history of traumatic brain injury (TBI) has been considered a risk factor for Alzheimer dementia. However, the specific association of TBI, even without loss of consciousness (LOC), with pathologic findings that underlie Alzheimer dementia, including Alzheimer disease (AD), non-AD neurodegenerative, and vascular pathologic findings, remains unclear.

**Objective:**

To examine the association between TBI with and without LOC and neuropathologic findings in community-based cohorts.

**Design, Setting, and Participants:**

This cross-sectional analysis used neuropathologic data from 1689 participants from the Religious Orders Study, the Rush Memory and Aging Project, and the Minority Aging Research Study. These studies began enrollment in 1994, 1997, and 2004, respectively. The current study’s data set was frozen on April 3, 2021, when the mean (SD) length of follow-up for the participants was 8.7 (5.5) years.

**Exposure:**

Traumatic brain injury exposure was assessed using a standardized, self-reported questionnaire at baseline and annual follow-up visits. Participants were categorized into those (1) without TBI exposure (n = 1024), (2) with TBI with LOC (n = 161), or (3) with TBI without LOC (n = 504).

**Main Outcomes and Measures:**

Neuropathologic measures of amyloid-β, paired helical filament tangles, neocortical Lewy bodies, transactive response DNA-binding protein 43, hippocampal sclerosis, gross infarcts, and microinfarcts were assessed. Multiple linear regression and logistic regression models were used to determine whether TBI with or without LOC (compared with no TBI exposure as the reference group) was associated with neuropathologic outcomes after adjusting for age at death, sex, and educational level. Whether the apolipoprotein E (*APOE*) ε4 allele and sex differences modified associations was also examined.

**Results:**

A total of 1689 participants (1138 [67%] women and 551 [33%] men; mean [SD] age at death, 89.2 [6.7] years; 80 [5%] Black, 46 [3%] Latino, 1639 [97%] non-Latino, and 1601 [95%] White) participated in the study. Compared with participants without TBI, participants with TBI with LOC had a greater amyloid-β load (estimate, 0.25; 95% CI, 0.06-0.43; *P* = .008) and higher odds of having 1 or more gross infarcts (odds ratio [OR], 1.45; 95% CI, 1.04-2.02; *P* = .02) and 1 or more microinfarcts (OR, 1.70; 95% CI, 1.21-2.38; *P* = .002), particularly subcortical microinfarcts (OR, 1.85; 95% CI, 1.23-2.79; *P* = .002). Those with TBI without LOC had higher odds of neocortical Lewy bodies (OR, 1.37; 95% CI, 1.01-1.87; *P* = .04) and 1 or more cortical microinfarcts (OR, 1.43; 95% CI, 1.09-1.87; *P* = .008). The association of TBI with and without LOC with vascular pathologic outcomes persisted after controlling for vascular risk factors and vascular disease burden. Traumatic brain injury with or without LOC was not associated with paired helical filament tangles, transactive response DNA-binding protein 43, or hippocampal sclerosis. No interactions occurred with *APOE* ε4 or sex.

**Conclusions and Relevance:**

This cross-sectional analysis suggests that a history of TBI, even without LOC, is associated with age-related neuropathologic outcomes, both neurodegenerative and vascular. The variation in the neuropathologic outcomes in individuals with and without LOC may provide clues to potential mechanisms, diagnoses, and management in persons with TBI.

## Key Points

**Question:**

Is traumatic brain injury (TBI) with and without loss of consciousness (LOC) associated with dementia-related neuropathologic outcomes in older persons?

**Findings:**

In this cross-sectional analysis of 1689 autopsied participants from 3 community-based cohort studies, participants with TBI with LOC had greater amyloid-β burden and higher odds of gross infarcts and microinfarcts, whereas those with TBI without LOC had higher odds of neocortical Lewy bodies and cortical microinfarcts.

**Meaning:**

Even without LOC, TBI may be associated with higher odds of neurodegenerative and vascular pathologic findings in later life.

## Teaser

This cross-sectional study of autopsied participants assesses whether traumatic brain injury (TBI) with and without loss of consciousness is associated with dementia-related neuropathologic outcomes in older persons.

## Introduction

Traumatic brain injury (TBI) is associated with substantial morbidity and mortality and constitutes a common and devastating health problem in adults. Moderate to severe TBI is an established risk factor for cognitive impairment and dementia,^1,2,3^ especially in those with genetic risk factors, such as apolipoprotein E (*APOE*) ε4 alleles.^4,5,6^ However, findings for mild TBI in relation to dementia^3,7,8^ are more mixed. Clinicopathologic studies have been crucial in advancing knowledge and providing new insights regarding the association between TBI and Alzheimer disease (AD) and other age-related neuropathologic conditions to understand the pathologic mechanism by which TBI may increase the risk of dementia. To date, most data are derived from clinic-based and autopsy-based samples and may not reflect TBI in community-based samples.^9,10,11^ Furthermore, there are few data on TBI with loss of consciousness (LOC) and neuropathologic outcomes in community-based cohorts^12,13,14^ and even less on the association of TBI without LOC and neuropathologic outcomes.

In the current study, we examine associations between TBI with and without LOC and neuropathologic outcomes by linking self-reported TBI with neuropathologic data from community-dwelling older persons enrolled in community-based studies. This study uses data from the autopsied brains of 1689 participants to examine whether self-reported TBI with and without LOC is associated with amyloid-β, paired helical filament (PHF) tangles, neocortical Lewy bodies (LBs), gross infarcts, microinfarcts, and other neuropathologic changes in the aging brain.

## Methods

### Participants

Older participants without known dementia were enrolled in 1 of 3 longitudinal, community-based cohort studies of aging and cognition: the Religious Orders Study, the Rush Memory and Aging Project, or the Minority Aging Research Study.^15,16^ Race and ethnicity were reported by participants at the baseline evaluation; these variables were included because of their importance to the risks, incidence, and consequences of common chronic diseases among older people. All 3 studies and the current cross-sectional study were approved by the Rush Institutional Review Board of Rush University Medical Center. Each participant signed a consent form agreeing to annual clinical evaluations and an Anatomical Gift Act form agreeing to autopsy and organ donation at the time of death. Organ donation was optional for participants enrolled in the Minority Aging Research Study. This study followed the Strengthening the Reporting of Observational Studies in Epidemiology (STROBE) reporting guideline for cross-sectional studies.

The Religious Orders Study started in 1994 with rolling enrollment, and 1495 participants have enrolled to date, of whom 872 died and 803 were autopsied; 790 with neuropathologic data were eligible for this study. The Rush Memory and Aging Project began in 1997 with 2210 participants enrolled to date, of whom 1079 died and 880 were autopsied; 866 with neuropathologic data were included. The Minority Aging Research Study started in 2004 and has enrolled 791 participants to date, of whom 196 have died and 35 were autopsied; 33 participants had neuropathologic data and were included. At the time of first analysis conducted on April 3, 2021, data from 1689 participants with baseline assessment data on brain injury and neuropathologic findings were available.

### TBI Assessment

Traumatic brain injury was assessed at enrollment and annual follow-up visits (mean [SD] follow-up, 8.7 [5.5] years) across all 3 studies based on the structured questionnaire.^12^ In brief, each participant was asked 2 questions to identify the presence and absence of TBI with or without LOC: (1) whether they have ever had a brain injury, and if so, (2) whether they ever experienced LOC resulting from brain injury. For analyses, decedents were categorized as (1) having no history of head injury (the reference category), (2) having TBI with LOC, or (3) having TBI without LOC. Those who reported 1 or more TBIs, at least 1 of which was with LOC, were considered members of the second group.

### Neuropathologic Evaluations

Brain autopsies (median postmortem interval, 6.91 hours; IQR, 5.01-10.50 hours) and fixation were performed by trained staff following a standard protocol.^17^ Uniform neuropathologic evaluations assessed amyloid-β burden, PHF tangle density, neocortical LBs, Parkinson disease (PD) pathologic findings, limbic-predominant age-related transactive response DNA-binding protein 43 encephalopathy neuropathologic change (LATE-NC), hippocampal sclerosis (HS), and the presence and location of cerebral gross infarcts and microinfarcts, categorized as subcortical vs cortical. Details of the pathologic assessment are provided in the eMethods in the Supplement and in prior studies.^18,19,20,21,22^ Methods for other covariates (demographics, *APOE* alleles, and vascular risk factors and diseases) are provided in the eMethods in the Supplement.

### Statistical Analysis

Groups (no TBI, TBI with LOC, and TBI without LOC) were compared by demographic, clinical, and neuropathologic characteristics using χ^2^ tests for categorical variables and analysis of variance for continuous variables followed by Tukey-Kramer test to compare pairs of TBI groups. Next, we conducted multivariable linear and logistic regressions to examine whether 2 TBI exposure groups (TBI with LOC and TBI without LOC compared with no TBI exposure as the reference group) were associated with neuropathologic outcomes, adjusted for age at death, sex, and educational level (reference model). We conducted linear regression models for overall measures of amyloid-β and PHF neurofibrillary tangle density comparing TBI with and without LOC with no TBI. Because a prior study^23^ reported that TBI may contribute to AD pathologic markers in some brain regions more than others, we further examined the association within the mesial temporal and separately within the neocortex regions.

Binary or ordinal logistic regression models were used separately for pathologic diagnosis of AD, PD, neocortical LBs, LATE-NC stage, HS, and the 3-level measure of both total gross infarcts and total microinfarcts. In separate models, we repeated the ordinal logistic regression for cortical and subcortical gross infarcts and separately for cortical and subcortical microinfarcts to determine whether the association of TBI with and without LOC with gross infarcts and microinfarcts differed by anatomical location. Because other vascular conditions might confound the associations of TBI with LOC and gross infarcts and microinfarcts,^24^ we repeated models with significant association with infarcts while adding terms for vascular risk factors and diseases.

Given previous reports^5,6,23^ of differences based on *APOE* ε4 and sex in the association of TBI with neuropathologic findings, we conducted 2 additional analyses by augmenting models with significant associations between TBI and a neuropathologic outcome: (1) one analysis in which we added 3 terms, one for *APOE* ε4 and the pair of terms for interaction of *APOE* ε4 with each TBI exposure; and (2) another analysis in which we added the pair of terms for interaction of male sex with each TBI exposure.

All analyses were programmed in SAS/STAT software, version 9.4 (SAS Institute Inc). A nominal threshold of 2-sided *P* < .05 for statistical significance was used.

## Results

A total of 1689 participants (1138 [67%] women and 551 [33%] men; mean [SD] age at death, 89.2 [6.7] years; 80 [5%] Black, 46 [3%] Latino, 1639 [97%] non-Latino, and 1601 [95%] White) were classified by TBI group: (1) no TBI (n = 1024), (2) TBI with LOC (n = 161), and (3) TBI without LOC (n = 504) (eFigure in the Supplement). Because some of the pathologic measures, such as LATE-NC, were introduced years after the first case was collected, a few participants had incomplete neuropathologic data; complete neuropathologic measures were available for 1578 of 1689 decedents. Information on demographic, *APOE* ε4, and neuropathologic data by the TBI group are given in Table 1.

**Table 1.** Demographic, Clinical, and Neuropathologic Characteristics of the Study Participants^a^

**Characteristic**	**All participants (N = 1689)**	**No TBI (n = 1024)**	**TBI with LOC (n = 161)**	**TBI without LOC (n = 504)**	***P* value**
**Demographic characteristics**					
Age at death, mean (SD), y	89.2 (6.7)	89.0 (6.7)	89.5 (7.7)	89.6 (6.4)	.26
Sex					
Female	1138 (67)	681 (66)	97 (60)	360 (71)	.02
Male	551 (33)	343 (33)	64 (40)	144 (29)	
Race					
Black	80 (5)	54 (5)	2 (1)	24 (5)	.08
White	1601 (95)	968 (95)	157 (99)	476 (95)	
Ethnicity					
Latino	46 (3)	26 (3)	7 (4)	13 (3)	.41
Non-Latino	1639 (97)	996 (97)	154 (96)	489 (97)	
Educational level, mean (SD), y	16.2 (3.6)	16.0 (3.5)	16.6 (3.7)	16.6 (3.8)	.004
**Clinical characteristics**					
Apolipoprotein E ε4^b^	428 (26)	270 (27)	43 (27)	115 (24)	.40
Burden of vascular risk factors at last evaluation, mean (SD)^c^	1.07 (0.85)	1.06 (0.85)	1.20 (0.87)	1.05 (0.82)	.12
Hypertension	1127 (67)	662 (65)	119 (74)	346 (69)	.03
Diabetes	363 (21)	214 (21)	37 (23)	112 (22)	.74
Smoking history	532 (32)	321 (31)	59 (37)	152 (30)	.29
Burden of vascular disease at last evaluation, mean (SD)^c^	0.69 (0.78)	0.65 (0.76)	0.76 (0.79)	0.76 (0.82)	.01
History of stroke	354 (21)	203 (20)	35 (22)	116 (23)	.35
History of myocardial infarction	352 (21)	198 (19)	38 (24)	116 (23)	.16
History of claudication in lower limbs	465 (28)	262 (26)	50 (31)	153 (31)	.08
**Neuropathologic characteristics**					
Square root of amyloid-β load, mean (SD)	1.54 (1.12)	1.5 (1.13)	1.74 (1.15)	1.58 (1.08)	.03
Square root of τ-tangles burden, mean (SD)	1.62 (1.36)	1.62 (1.36)	1.47 (1.30)	1.67 (1.37)	.25
AD pathologic diagnosis	1081 (64)	644 (63)	105 (65)	332 (66)	.49
PD pathologic diagnosis^d^	139 (8)	81 (8)	13 (8)	45 (9)	.83
Neocortical LBs^e^	224 (13)	120 (12)	27 (17)	77 (15)	.06
LATE-NC stage^f^					
0	759 (48)	449 (48)	82 (53)	228 (47)	.72
1	293 (19)	177 (19)	23 (15)	93 (19)	
2	156 (10)	97 (10)	16 (10)	43 (9)	
3	368 (23)	215 (23)	33 (21)	120 (25)	
Hippocampal sclerosis^g^	160 (9)	89 (9)	56 (11)	15 (9)	.32
Gross infarcts					
No infarct	1090 (64)	680 (66)	93 (58)	317 (63)	.08
1 Infarct	312 (18)	176 (17)	31 (19)	105 (21)	
Multiple infarcts	287 (17)	168 (16)	37 (23)	82 (16)	
Microinfarcts					
No infarct	1178 (70)	738 (72)	97 (60)	343 (68)	.01
1 Infarct	304 (18)	179 (17)	36 (22)	89 (18)	
Multiple infarcts	207 (12)	107 (10)	28 (17)	72 (14)	

Abbreviations: AD, Alzheimer disease; LATE-NC, limbic-predominant age-related transactive response DNA-binding protein 43 encephalopathy neuropathologic change; LBs, Lewy bodies; LOC, loss of consciousness; PD, Parkinson disease; TBI, traumatic brain injury.

^a^ Data are presented as number (percentage) of participants unless otherwise indicated.

^b^ Measures not assessed for 32 participants (3 in the TBI with LOC group, 17 in the TBI without LOC group, and 12 in the non-TBI group).

^c^ Measures not assessed for 34 participants (1 in the TBI with LOC group, 13 in the TBI without LOC group, and 20 in the non-TBI group).

^d^ Measures not assessed for 54 participants (8 in the TBI with LOC group, 11 in the TBI without LOC group, and 35 in the non-TBI group).

^e^ Measures not assessed for 1 participant (in the non-TBI group).

^f^ Measures not assessed for 113 participants (7 in the TBI with LOC group, 20 in the TBI without LOC group, and 86 in the non-TBI group).

^g^ Measures not assessed for 11 participants (2 in the TBI with LOC group, 2 in the TBI without LOC group, and 7 in the non-TBI group).

Male participants were more likely to report no TBI (343 of 551 men [62.3%]) and TBI with LOC (64 of 551 men [11.6%]) than female participants (681 of 1138 [59.8%] and 97 of 1138 [8.5%]), respectively. The TBI groups differed by educational level (Table 1), with participants with TBI without LOC reporting more years of education than the non-TBI group (estimate, 0.60; 95% CI, 0.13-1.06; *P* = .006).

### Associations of TBI Group With AD and Other Neurodegenerative Pathologic Findings

The mean (SD) square root–transformed amyloid-β load was 1.54 (1.12), with the 90th percentile being 3 units. The mean (SD) square root–transformed PHF neurofibrillary tangle density was 1.62 (1.36), with the 90th percentile being 3.6 units in all participants. For AD pathologic findings, only the amyloid-β load differed by TBI group, with the TBI with LOC group having a higher amyloid-β load than the non-TBI group (estimate, 0.23; 95% CI, 0.01-0.46; *P* = .03); no differences were found when comparing the TBI without LOC group with the non-TBI group (estimate, 0.07; 95% CI, −0.06 to 0.22; *P* = .41) and comparing the TBI with LOC group with the TBI without LOC group (estimate, 0.16; 95% CI, −0.08 to 0.40; *P* = .26).

Findings were similar after adjusting for age at death, sex, and educational level (Table 2). In linear regression models, TBI with LOC was associated with higher overall amyloid-β load (estimate, 0.25; 95% CI, 0.06-0.43; *P* = .008) than non-TBI; TBI without LOC was not associated with amyloid-β load. Both TBI with and without LOC were not associated with neurofibrillary tangle burden (Table 2).

**Table 2.** Association of TBI With and Without LOC With AD and Other Non-AD Neurodegenerative Outcomes^a^

Outcome	TBI with LOC (n = 161)		TBI without LOC (n = 504)	
	Estimate or OR (95% CI)	*P* value	Estimate or OR (95% CI)	*P* value
**Continuous**				
Square root of amyloid-β burden				
Overall	0.25 (0.06 to 0.43)	.008	0.07 (−0.05 to 0.19)	.27
Neocortical	0.25 (0.06 to 0.45)	.01	0.05 (−0.07 to 0.18)	.40
Mesial temporal	0.23 (0.06 to 0.39)	.007	0.10 (−0.01 to 0.20)	.07
Square root of τ-tangles burden				
Overall	−0.14 (−0.36 to 0.09)	.23	0.03 (−0.01 to 0.18)	.65
Neocortical	−0.09 (−0.31 to 0.13)	.42	0.02 (−0.12 to 0.16)	.76
Mesial temporal	−0.30 (−0.61 to 0.02)	.06	0.03 (−0.61 to 0.02)	.74
**Categorical**				
AD pathologic diagnosis	1.10 (0.77 to 1.58)	.59	1.08 (0.86 to 1.362)	.50
PD pathologic diagnosis	1.02 (0.55 to 1.88)	.95	1.14 (0.78 to 1.68)	.49
Neocortical Lewy bodies	1.50 (0.95 to 2.37)	.08	1.37 (1.01 to 1.87)	.04
LATE-NC stage				
0-3	0.78 (0.56 to 1.09)	.14	1.01 (0.82 to 1.25)	.90
≥2	0.91 (0.62 to 1.32)	.61	1.00 (0.79 to 1.27)	.99
Hippocampal sclerosis	1.05 (0.59 to 1.87)	.87	1.29 (0.90 to 1.84)	.16

Abbreviations: AD, Alzheimer disease; LATE-NC, limbic-predominant age-related transactive response DNA-binding protein 43 encephalopathy neuropathologic change; LOC, loss of consciousness; OR, odds ratio; PD, Parkinson disease; TBI, traumatic brain injury.

^a^ All models were adjusted for age at death, sex, and educational level. Continuous variables are presented as estimates (95% CIs) and categorical variables as ORs (95% CIs).

Similar to overall amyloid-β load, TBI with LOC had a higher mesial temporal amyloid-β load (estimate, 0.23; 95% CI, 0.06-0.39; *P* = .007) and neocortical amyloid-β load (estimate, 0.25; 95% CI, 0.06-0.45; *P* = .01) than those without TBI after adjusting for age, sex, and educational level, but TBI without LOC was not associated with regional measures of amyloid-β. No association was found of TBI exposure group with PHF neurofibrillary tangles in region or pathologic diagnosis of AD (Table 2).

For other non-AD neurodegenerative pathologic findings, those with TBI without LOC had increased odds of having neocortical LBs (odds ratio [OR], 1.37; 95% CI, 1.01-1.87; *P* = .04) after adjusting for demographic characteristics. We found no association between TBI with LOC and neocortical LBs (OR, 1.50; 95% CI, 0.95-2.37; *P* = .08) (Figure, A). The TBI exposure group was not associated with pathologic diagnoses of PD, LATE-NC, and HS (Table 2).

**Figure.**
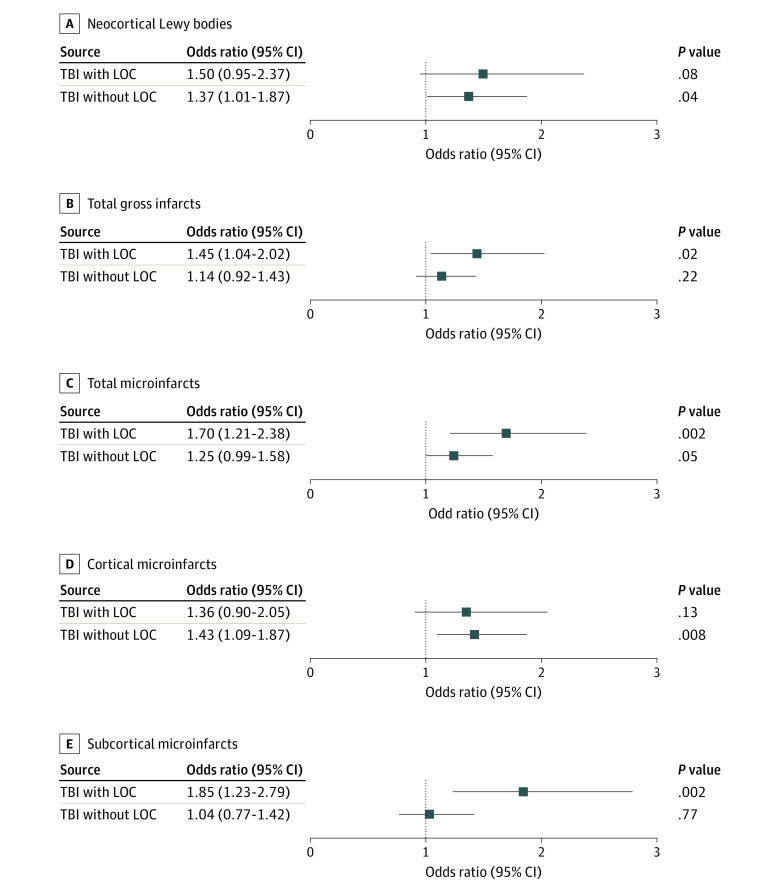
Odds Ratios Provided for Neuropathologic Outcomes Neuropathologic outcomes included neocortical Lewy bodies, total gross infarcts, total microinfarcts, cortical microinfarcts, and subcortical microinfarcts. Odds ratios are provided for traumatic brain injury (TBI) with loss of consciousness (LOC) and TBI without LOC compared with not having TBI exposure from models adjusted for age, sex, and educational level. Error bars indicate 95% CIs.

### Associations of TBI Group With Cerebral Infarcts

A series of ordinal logistic regression models was conducted to examine the association of TBI group with gross infarcts and with microinfarcts, controlling for age at death, sex, and educational level. The TBI groups differed in odds of gross infarcts and microinfarcts, with the TBI with LOC group more likely to have 1 or more gross infarcts (OR, 1.45; 95% CI, 1.04-2.02; *P* = .02) and microinfarcts (OR, 1.70; 95% CI, 1.21-2.38; *P* = .002) (Table 3 , Table 4, and Figure); the TBI without LOC group did not have elevated odds of infarcts.

**Table 3.** Association of TBI With and Without LOC With Gross Infarcts^a^

Outcome and model set	TBI with LOC (n = 161)		TBI without LOC (n = 504)	
	OR (95% CI)	*P* value	OR (95% CI)	*P* value
Gross infarcts, total				
1	1.45 (1.04-2.02)	.02	1.14 (0.92-1.43)	.22
2	1.44 (1.03-2.00)	.03	1.14 (0.91-1.42)	.25
3	1.44 (1.03-2.02)	.03	1.10 (0.88-1.38)	.38
4	1.42 (1.02-1.98)	.03	1.10 (0.88-1.38)	.39
Gross infarcts, cortical				
1	1.48 (0.95-2.30)	.08	1.04 (0.76-1.43)	.80
2	1.47 (0.94-2.30)	.08	1.04 (0.75-1.44)	.81
3	1.42 (0.91-2.22)	.12	0.98 (0.71-1.36)	.90
4	1.42 (0.90-2.22)	.12	0.98 (0.71-1.36)	.90
Gross infarcts, subcortical				
1	1.14 (0.79-1.64)	.48	1.10 (0.86-1.39)	.45
2	1.13 (0.78-1.63)	.51	1.09 (0.86-1.39)	.48
3	1.11 (0.77-1.60)	.58	1.05 (0.82-1.34)	.68
4	1.09 (0.76-1.58)	.63	1.05 (0.82-1.34)	.68

Abbreviations: LOC, loss of consciousness; OR, odds ratio; TBI, traumatic brain injury.

^a^ Model set 1 adjusted for age at death, sex, and educational level. In addition to model set 1 terms, model set 2 was additionally adjusted for sum of vascular risk factors, model set 3 for number of vascular diseases, and model set 4 for both the number of vascular risk factors and sum of vascular diseases.

**Table 4.** Association of TBI With and Without LOC With Microinfarcts^a^

Outcome and model set	TBI with LOC (n = 161)		TBI without LOC (n = 504)	
	OR (95% CI)	*P* value	OR (95% CI)	*P* value
Microinfarcts, total				
1	1.70 (1.21-2.38)	.002	1.25 (0.99-1.58)	.05
2	1.70 (1.21-2.38)	.002	1.24 (0.98-1.57)	.06
3	1.66 (1.18-2.34)	.003	1.22 (0.96-1.54)	.09
4	1.66 (1.18-2.33)	.003	1.22 (0.96-1.54)	.09
Microinfarcts, cortical				
1	1.36 (0.90-2.05)	.13	1.43 (1.09-1.87)	.008
2	1.38 (0.91-2.08)	.12	1.41 (1.07-1.85)	.01
3	1.35 (0.89-2.03)	.15	1.39 (1.06-1.82)	.01
4	1.35 (0.90-2.04)	.14	1.39 (1.06-1.82)	.01
Microinfarcts, subcortical				
1	1.85 (1.23-2.79)	.002	1.04 (0.77-1.42)	.77
2	1.83 (1.22-2.75)	.003	1.04 (0.76-1.42)	.78
3	1.81 (1.20-2.73)	.004	1.02 (0.75-1.39)	.89
4	1.79 (1.19-2.70)	.005	1.02 (0.75-1.39)	.89

Abbreviations: LOC, loss of consciousness; OR, odds ratio; TBI, traumatic brain injury.

^a^ Model set 1 adjusted for age at death, sex, and educational level. In addition to model set 1 terms, model set 2 was additionally adjusted for sum of vascular risk factors, model set 3 for number of vascular diseases, and model set 4 for both the number of vascular risk factors and sum of vascular diseases.

To further examine whether the association with gross infarcts differs by anatomical locations, we fit separate logistic regression models for cortical and subcortical gross infarcts and microinfarcts. The TBI group was not significantly associated with cortical or subcortical gross infarcts (Table 3 and Figure). For cortical or subcortical microinfarcts, the TBI group was associated with localized microinfarcts, with the TBI with LOC group having higher odds of 1 or more subcortical microinfarcts (OR, 1.85; 95% CI, 1.23-2.79; *P* = .002) and the TBI without LOC group having 1 or more cortical microinfarcts (OR, 1.43; 95% CI, 1.09-1.87; *P* = .008) (Table 4).

In sensitivity analyses, we found that the association of the TBI group with infarcts remained unchanged after controlling for vascular risk factors and diseases (Table 3 and Table 4). We also examined whether the association of the TBI exposure group with neuropathologic outcomes persisted after adjustment for study cohort (Religious Orders Study vs other cohorts); significant associations were retained (eTable in the Supplement). Finally, *APOE* ε4 and, separately, sex were included as interaction terms with TBI exposure (with and without LOC) in separate models of amyloid-β load, neocortical LBs, gross infarcts, and microinfarcts; no significant interactions terms were found in any model.

## Discussion

In this cross-sectional study, the brains of older persons with a history of TBI with LOC and, separately, TBI without LOC were found to have increased neurodegenerative and vascular pathologic findings. For neurodegenerative pathologic findings, individuals with TBI with LOC exhibited a higher burden of overall amyloid-β, whereas those without LOC showed an increased odds of the presence of neocortical LBs. For brain vascular pathologic findings, TBI with LOC had increased risk for 1 or more gross infarcts and microinfarcts, specifically, for subcortical microinfarcts, whereas TBI without LOC had higher odds of 1 or more cortical microinfarcts. Our study extends previous community-based studies^12,14^ of TBI and neuropathologic findings in several important ways. First, we evaluated molecular markers of AD pathologic findings and found that TBI with LOC was associated with brain amyloid-β deposition but not neuronal PHF-τ tangles. Second, we found that TBI without LOC, similar to our findings with TBI with LOC, was associated with neocortical LBs. Third, we found that TBI with and TBI without LOC were associated with brain infarcts, with TBI without LOC most notably associated with cortical microinfarcts. Overall, these findings suggest that not only TBI with LOC but also TBI without LOC may increase susceptibility to late-life neurodegenerative and vascular brain pathologic outcomes associated with dementia.

Few previous studies^12,14^ have examined the association between the history of TBI with LOC and neuropathologic changes in community populations, and these studies had mixed results. A previous study^12^ reported an association of TBI with LOC with neocortical LBs and microinfarcts using data from the Religious Orders Study, Rush Memory and Aging Project, and Adult Changes in Thought cohorts; we did not find any association with neuritic plaques, but continuous measures of cortical amyloid load were not available for both cohort studies. Other studies^10,14^ have also shown no differences in neuritic plaques between people with and without a history of TBI but failed to confirm the association between TBI with LOC and neocortical LBs or microinfarcts. Moreover, a prior study^23^ found an association between TBI with LOC and neuritic plaques in men, although other neuropathologic measures, such as LBs and infarcts, were not studied. We observe a higher accumulation of neocortical and mesial temporal amyloid-β load in the respondents with a history of TBI with LOC. Our result is consistent with a prior neuroimaging study^25^ that showed that among people with mild cognitive impairment, amyloid deposition was greater in persons with TBI with LOC compared with persons without TBI with LOC. Considerable evidence suggests that amyloid is an early marker in the development of AD, appearing to begin in the neocortex and affecting the mesial temporal lobe in advanced age.^26,27^ Despite the association of TBI with LOC with neocortical and mesial temporal amyloid—the sine qua non for Alzheimer neuropathologic changes—reported herein, we did not find an association of TBI with pathologic diagnosis of AD. However, a specific association of TBI with LOC, but not without LOC, with amyloid-β load in 2 key regions, but not with a neurofibrillary tangle burden, suggests that severity of TBI may be important for the development of amyloid pathologic outcomes.

A previous study^12^ reported that respondents with TBI had increased odds of neocortical LBs. The current study found that this association was present for those with TBI with no LOC. We also found 40% higher odds of neocortical LBs in people with TBI without LOC. Unlike in the previous study,^12^ the statistical evidence is weaker for the TBI with LOC group, although the important point is that the estimated OR for TBI with LOC is higher (OR, 1.50) than TBI without LOC (OR, 1.37). In addition, the TBI with LOC group had wider CIs because there were fewer people in the TBI with LOC group. A future study with more participants with TBI with LOC is warranted to further establish the association of TBI with LOC and neocortical LBs.

Cerebral infarcts were frequent, and our findings are consistent with the previous finding of higher odds of microinfarcts among those with TBI with LOC.^12^ We specifically show 85% higher odds of having subcortical microinfarcts in those with TBI with LOC. The current study newly reports that TBI with LOC was associated with a higher likelihood of gross infarcts, specifically an approximately 40% increase in odds. An important novel finding is the association of TBI without LOC with microinfarcts. We specifically found 43% higher odds of cortical microinfarcts in those with TBI without LOC. All of these findings were robust and remained significant when controlling for vascular risk factors and vascular diseases. Prior studies^28,29,30,31^ have shown that differential structural and pathologic changes can occur in cortical and deep subcortical regions after brain injury.

Several studies^4,5,6,32^ have described *APOE* ε4 risk alleles and sex as risk factors for neuropathologic outcomes in people with TBI and showed that the associations of TBI with dementia or AD-related pathologic outcomes are greater in people with *APOE* ε4 risk alleles than in non–*APOE* ε4 carriers and in men than in women,^23^ whereas some studies^12,33^ have found no differences. Consistent with previous findings,^12^ this study did not demonstrate different associations by *APOE* ε4 groups or by sex. Additional studies are needed to determine whether other potential risk factors (eg, sleep disturbance, posttraumatic stress, or depression) influence the association of TBI with neuropathologic outcomes.^34,35,36^

### Strengths and Limitations

This study has several strengths. The current study expanded on previous work by including a milder form of TBI (TBI without LOC) and using a community-based (rather than a clinic-based) sample. The large sample size of the current autopsy cohort, made possible by uniform data collection across 3 cohorts, provides adequate statistical power to investigate multiple pathologic end points in adjusted models. Finally, some potential confounders, such as vascular risk factors and disease burden, were adjusted to assess the robustness of TBI and vascular findings.

The study also has some limitations. The participants were relatively highly educated and had a mean age of death greater than the national mean for age of death; thus, these findings may not generalize to the younger population and persons with a wider range of educational experiences. Because TBI data are self-reported, recall bias may have resulted in case misclassification. However, our cohorts were enrolled without dementia, possibly attenuating this potential bias. The pathologic basis of TBI is complicated and dependent on numerous factors, including length of the LOC, frequency, and age at injury; these factors may influence neuropathologic results. For example, only 13% of our sample reported more than 1 lifetime TBI with or without LOC; therefore, we could not examine the role of repeated TBI. Future studies in different populations with detailed characterization of TBI will be important to understand how variations in these factors influence the differential neuropathologic presentation of the different types of neurodegenerative disorders. Our PHF tangle density measure describes only neuronal PHF tangle burden but not perivascular neuronal τ or astrocytic τ because we do not have complete pathologic data on chronic traumatic encephalopathy and age-related τ astrogliopathy. Investigating the association of TBI with chronic traumatic encephalopathy and age-related τ astrogliopathy pathologic outcomes would be a relevant topic for future studies because emerging studies^37,38^ have shown that repetitive head impact from contact sports or military combat is associated with chronic traumatic encephalopathy.

## Conclusions

The results of this cross-sectional study lead to several conclusions that may apply to the general population. First, TBI with LOC may be associated with greater amyloid-β burden and vascular brain pathologic outcomes. Second, TBI even without LOC may have long-term neurodegenerative and vascular consequences. These data provide support for the hypothesis that TBI with and even without LOC may be important risk factors for late-life cognitive and motor impairment in aging. Finally, the variation in the neuropathologic outcomes in individuals with and without LOC may provide clues to potential mechanisms, diagnoses, and management in persons with TBI.

## Notes

Supplement. **eMethods.** Assessment of Dementia-Related Pathologies **eFigure.** Overview of the Study Design **eTable.** Association of TBI With and Without LOC With Neurodegenerative and Vascular Pathology Indices Adjusted by Demographics and Study Cohort
