# Supplementary material for: Association of Traumatic Brain Injury With and Without Loss of Consciousness With Neuropathologic Outcomes in Community-Dwelling Older Persons
Source: JAMA Netw Open. 2022 Apr 27;5(4):e229311. doi: 10.1001/jamanetworkopen.2022.9311 (PMC9047640; doi:10.1001/jamanetworkopen.2022.9311)
Supplement: Supplement. — eMethods. Assessment of Dementia-Related Pathologies eFigure. Overview of the Study Design eTable. Association of TBI With and Without LOC With Neurodegenerative and Vascular Pathology Indices Adjusted by Demographics and Study Cohort [file jamanetwopen-e229311-s001.pdf]

# Supplementary Online Content

Agrawal S, Leurgans SE, James BD, et al. Association of traumatic brain injury with and without loss of consciousness with neuropathologic outcomes in community-dwelling older persons. *JAMA Netw Open*. 2022;5(4):e229311. doi:10.1001/jamanetworkopen.2022.9311

**eMethods.** Assessment of Dementia-Related Pathologies

**eFigure.** Overview of the Study Design

**eTable.** Association of TBI With and Without LOC With Neurodegenerative and Vascular

Pathology Indices Adjusted by Demographics and Study Cohort

**This supplementary material has been provided by the authors to give readers additional information about their work.**

© 2022 Agrawal S et al. *JAMA Network Open.*

eMethods. Assessment of Dementia-Related Pathologies

**Post-mortem Evaluations**

The autopsied brain was hemisected and cut into 1 cm coronal slabs. Slabs from one hemisphere were fixed in frozen in -80 degree Celsius freezers, and slabs from the other hemisphere were fixed in 4% paraformaldehyde and stored in 4 degree Celsius refrigerators. The neuropathologic evaluations were supervised by board-certified neuropathologists blinded to clinical information.

**Alzheimer's disease pathology**

Amyloid-beta (Aβ) was detected with immunohistochemistry from 8 brain regions (midfrontal, superior frontal, anterior cingulate, inferior temporal, entorhinal, angular gyrus, and calcarine cortices and hippocampus) using three monoclonal antibodies (10D5-1:600; Elan Pharmaceuticals, San Francisco, CA, 6F/3D-1:50; Dako North America Inc., Carpinteria, CA, and 4G8-1:9000; Covance Labs, Madison, WI) specific for Aβ. A systematic random sampling scheme was used to capture images for quantitative analysis of Aβ deposition and a composite continuous summary measure of the total amyloid-beta load was generated by the mean of the square root of the percent area of the region occupied by Aβ[1].

Paired helical filament (PHF)-tau was detected with anti-phospho tau AT8 antibody (Innogenetics, Alpharetta GA, 1:1000) from the same 8 above-mentioned brain regions, quantified as mean tangle density per $mm^2$ with a computerized sampling method, and generated a composite summary measure of the density of PHF-neurofibrillary tangle by averaging the values for all regions[1]. Square root transformation was applied to tau tangle density.

Data on PHF-neurofibrillary tangles and amyloid-beta load in each of the hippocampus and entorhinal cortex were combined to create a mesial temporal score for PHF-neurofibrillary tangle density and amyloid-beta load respectively, while data in the midfrontal cortex, superior frontal, angular gyrus, inferior temporal, and calcarine were combined to create a neocortical

© 2022 Agrawal S et al. *JAMA Network Open.*

score for PHF-neurofibrillary tangle density and amyloid-beta load[1, 2]. Square root transformation was also applied to regional tau tangle density and amyloid-beta load.

Also, a pathologic diagnosis of AD was determined using the modified National Institute on Aging-Reagan criteria as previously described[3].

**Lewy Body Disease and Parkinson's disease pathology**

Lewy body (LB) pathology was identified with antibodies specific to alpha-synuclein using alkaline phosphatase as the chromogen from the midfrontal, middle temporal, inferior parietal, entorhinal, and anterior cingulate cortices, amygdala, and substantia nigra and the presence of neocortical LBs was determined based on the presence of LBs in any neocortical regions[4]. Nigral neuronal loss was assessed in the substantia nigra and a pathologic diagnosis of PD was determined based on moderate or severe nigral neuronal loss with the presence of nigral LBs[5].

**Limbic-predominant age-related TDP-43 encephalopathy neuropathologic changes pathology**

Limbic-predominant age-related TDP-43 encephalopathy neuropathologic changes (LATE-NC) pathology was assessed by immunohistochemistry using phosphorylated monoclonal TAR5P-1D3, pS409/410 antibodies (before 2015; Ascenion, Munich, Germany, dilution 1:100 and since 2015; Millipore Sigma, Burlington, MA, dilution 1:400) [6, 7]. For analysis, TDP-43 distribution was grouped into four distinct stages; stage 0 (no TDP-43 inclusions); stage 1 (TDP-43 inclusions in amygdala only); stage 2 (TDP-43 inclusions in entorhinal cortex, hippocampus CA1/subiculum, or dentate gyrus), and stage 3 (extend into any neocortical regions)[8].

**Hippocampal sclerosis**

Hippocampal sclerosis (HS) was systematically evaluated unilaterally from the mid-hippocampus. Since 2019, we also included anterior hippocampus bilaterally and determined HS as present based on the presence of severe neuronal loss and gliosis (>90%) in the CA1/subiculum sub-region of any of three hippocampal regions for analyses[9].

© 2022 Agrawal S et al. *JAMA Network Open.*

**Gross infarcts and microinfarcts:** The location of gross infarcts were determined based on gross examination and the final confirmation of the presence, age, and number (single or multiple) of gross infarcts (i.e. infarcts visible to the naked eye) were confirmed histologically from 6-micron sections which were stained with hematoxylin/eosin[10]. Microinfarcts (i.e. infarcts not visible to the naked eye) were identified from the diagnostic brain regions, including cortical, subcortical, midbrain, and cerebellum regions and age, number, and location were confirmed by microscopy [11]. For the analysis, only chronic gross infarcts and microinfarcts (lesions with cystic cavitation with few macrophages surrounded by fibrillary gliosis) were included, and both gross and microinfarcts were summarized as no infarct reported, 1 infarct, or multiple infarcts (>1 infarct). Additional measures considered the location of gross infarcts and microinfarcts, with cortical and subcortical gross infarcts and microinfarcts as separate outcomes[12].

**Other covariates:**

Age-at-death in years was calculated from birth date and date of death. Education (in years), sex, race (White American and African American), and ethnicity (Latinos and Non-Latinos) were self-reported by participants at enrollment. *APOE* alleles and genotypes were determined by sequencing rs429358 (codon 112) and rs7412 (codon 158) at exon 4 of the *APOE* gene using Polymorphic DNA Technologies (Alameda, CA)[13]. All participants in this study were asked every year about 3 chronic vascular diseases (stroke, heart attack, and claudication) and 3 vascular risk factors (diabetes, hypertension, smoking). Smoking history (current or past) was reported at enrollment. Myocardial infarction was defined as clinically diagnosed heart attack, coronary thrombosis, coronary occlusion, or myocardial infarction, as reported by the participant. Stroke was diagnosed by the physician on the basis of a uniform structured neurological examination and self-reported medical history. Claudication was determined based on the presence of leg pain while walking and to describe the location of the leg pain by the participant. Classification of diabetes and hypertension was based on self-report and medication review. For each

© 2022 Agrawal S et al. *JAMA Network Open.*

vascular risk factor/disease burden score, we calculated the number of conditions seen, as previously described[14].

eFigure 1: Overview of the study design. Flow chart shows subject inclusion/exclusion criteria used in this study and arrangement of the final sample size.

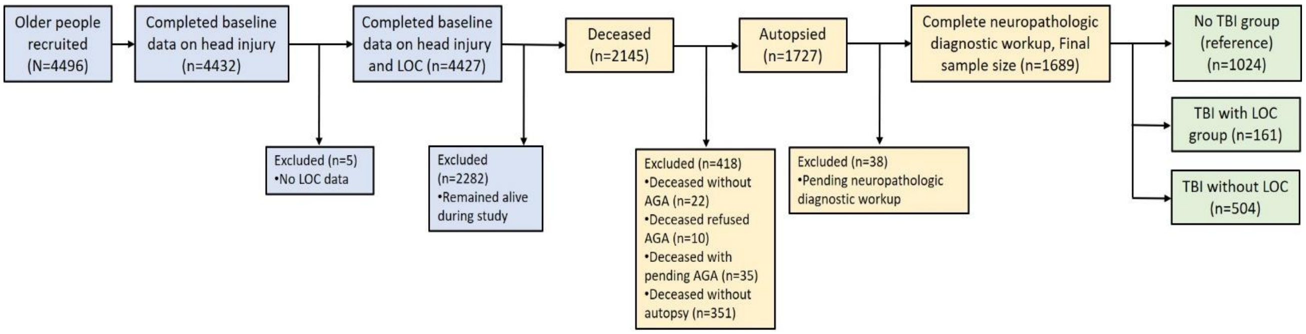


© 2022 Agrawal S et al. *JAMA Network Open.*

eTable 1: The association of TBI with and without LOC with AD and other non-AD neurodegenerative pathology indices adjusted by demographics and study cohort

| Outcome | | TBI with LOC (n=161) | | TBI without LOC (n=504) | |
|---|---|---|---|---|---|
| **Continuous** | | **Estimate (95% Confidence interval)** | **p-value** | **Estimate (95% Confidence interval)** | **p-value** |
| Square root of amyloid-beta burden | Overall | 0.266 (0.079 - 0.453) | 0.005 | 0.079 (-0.041 - 0.1991) | 0.19 |
| | Neocortical | 0.272 (0.077 - 0.466) | 0.006 | 0.066 (-0.058 - 0.191) | 0.29 |
| | Mesial temporal | 0.239 (0.071 - 0.406) | 0.005 | 0.106 (-0.01 - 0.212) | 0.06 |
| Square root of tau-tangles burden | Overall | -0.133 (-0.358 - 0.09) | 0.24 | 0.304 (-0.109 - 0.179) | 0.63 |
| | Neocortical | -0.086 (-0.171 - 0.116) | 0.43 | 0.022 (-0.306 - 0.132) | 0.75 |
| | Mesial temporal | -1.302 (-0.615 - 0.011) | 0.05 | 0.029 (-0.171 - 0.231) | 0.77 |
| **Categorical** | | **Odds Ratio (95% Confidence interval)** | **p-value** | **Odds Ratio (95% Confidence interval)** | **p-value** |
| AD pathologic diagnosis | | 1.091 (0.759 - 1.567) | 0.63 | 1.075 (0.853 - 1.354) | 0.53 |
| PD pathologic diagnosis | | 1.003 (0.542 - 1.856) | 0.99 | 1.133 (0.771 - 1.664) | 0.52 |
| Neocortical Lewy bodies | | 1.513 (0.957 - 2.389) | 0.07 | 1.382 (1.013 - 1.886) | 0.04 |
| LATE-NC (stage0-3) | | 0.787 (0.566 - 1.095) | 0.15 | 1.018 (0.827 - 1.253) | 0.86 |
| LATE-NC (stage≥2) | | 0.914 (0.627 - 1.331) | 0.63 | 1.004 (0.790 - 1.274) | 0.97 |
| Hippocampal sclerosis | | 1.007 (0.552 - 1.836) | 0.97 | 1.211 (0.836 - 1.754) | 0.30 |
| Gross infarcts | Total | 1.455 (1.045 - 2.024) | 0.02 | 1.143 (0.917 - 1.425) | 0.23 |
| | Cortical | 1.491 (0.957 - 2.321) | 0.07 | 1.046 (0.76 - 1.439) | 0.78 |
| | Subcortical | 1.131 (0.785 - 1.63) | 0.50 | 1.091 (0.859 - 1.384) | 0.47 |
| Microinfarcts | Total | 1.678 (1.197 - 2.353) | 0.002 | 1.243 (0.987 - 1.565) | 0.06 |

© 2022 Agrawal S et al. *JAMA Network Open.*

| | | | | | |
|---|---|---|---|---|---|
| | Cortical | 1.376 (0.914 - 2.071) | 0.125 | 1.439 (1.101 - 1.881) | 0.007 |
| | Subcortical | 1.815 (1.206 - 2.729) | 0.004 | 1.030 (0.757 - 1.401) | 0.84 |

The result indicated that the association of TBI with higher levels of amyloid-beta load and cerebral infarcts were not confounded by study cohorts.

© 2022 Agrawal S et al. *JAMA Network Open.*

**eReferences:**

1. Bennett DA, Schneider JA, Wilson RS, Bienias JL, Arnold SE. Neurofibrillary tangles mediate the association of amyloid load with clinical Alzheimer disease and level of cognitive function. *Arch Neurol*. 2004;61(3):378-384. doi: 10.1001/archneur.61.3.378[doi].

2. Schneider JA, Arvanitakis Z, Bang W, Bennett DA. Mixed brain pathologies account for most dementia cases in community-dwelling older persons. *Neurology*. 2007;69(24):2197-2204. doi: 01.wnl.0000271090.28148.24 [pii].

3. Hyman BT, Trojanowski JQ. Consensus recommendations for the postmortem diagnosis of Alzheimer disease from the National Institute on Aging and the Reagan Institute Working Group on diagnostic criteria for the neuropathological assessment of Alzheimer disease. *J Neuropathol Exp Neurol*. 1997;56(10):1095-1097. doi: 10.1097/00005072-199710000-00002 [doi].

4. Schneider JA, Arvanitakis Z, Yu L, Boyle PA, Leurgans SE, Bennett DA. Cognitive impairment, decline and fluctuations in older community-dwelling subjects with Lewy bodies. *Brain*. 2012;135(Pt 10):3005-3014. doi: 10.1093/brain/aws234 [doi].

5. Buchman AS, Shulman JM, Nag S, et al. Nigral pathology and parkinsonian signs in elders without Parkinson disease. *Ann Neurol*. 2012;71(2):258-266. doi: 10.1002/ana.22588 [doi].

6. Nag S, Yu L, Wilson RS, Chen EY, Bennett DA, Schneider JA. TDP-43 pathology and memory impairment in elders without pathologic diagnoses of AD or FTLD. *Neurology*. 2017;88(7):653-660. doi: 10.1212/WNL.0000000000003610 [doi].

7. Agrawal S, Yu L, Kapasi A, et al. Limbic-predominant age-related TDP-43 encephalopathy neuropathologic change and microvascular pathologies in community-dwelling older persons. *Brain Pathol*. 2021;31(3):e12939. doi: 10.1111/bpa.12939 [doi].

© 2022 Agrawal S et al. *JAMA Network Open.*

8. Nelson PT, Dickson DW, Trojanowski JQ, et al. Limbic-predominant age-related TDP-43 encephalopathy (LATE): consensus working group report. *Brain*. 2019;142(6):1503-1527. doi: 10.1093/brain/awz099 [doi].

9. Nag S, Yu L, Capuano AW, et al. Hippocampal sclerosis and TDP-43 pathology in aging and Alzheimer disease. *Ann Neurol*. 2015;77(6):942-952. doi: 10.1002/ana.24388 [doi].

10. Schneider JA, Wilson RS, Bienias JL, Evans DA, Bennett DA. Cerebral infarctions and the likelihood of dementia from Alzheimer disease pathology. *Neurology*. 2004;62(7):1148-1155. doi: 10.1212/01.wnl.0000118211.78503.f5 [doi].

11. Arvanitakis Z, Leurgans SE, Barnes LL, Bennett DA, Schneider JA. Microinfarct pathology, dementia, and cognitive systems. *Stroke*. 2011;42(3):722-727. doi: 10.1161/STROKEAHA.110.595082 [doi].

12. Arvanitakis Z, Capuano AW, Leurgans SE, Buchman AS, Bennett DA, Schneider JA. The Relationship of Cerebral Vessel Pathology to Brain Microinfarcts. *Brain Pathol*. 2017;27(1):77-85. doi: 10.1111/bpa.12365 [doi].

13. Yu L, Boyle PA, Nag S, et al. APOE and cerebral amyloid angiopathy in community-dwelling older persons. *Neurobiol Aging*. 2015;36(11):2946-2953. doi: S0197-4580(15)00419-4 [pii].

14. Bennett DA, Buchman AS, Boyle PA, Barnes LL, Wilson RS, Schneider JA. Religious Orders Study and Rush Memory and Aging Project. *J Alzheimers Dis*. 2018;64(s1):S161-S189. doi: 10.3233/JAD-179939 [doi].

© 2022 Agrawal S et al. *JAMA Network Open.*
